# Fatal canine distemper virus infection of giant pandas in China

**DOI:** 10.1038/srep27518

**Published:** 2016-06-16

**Authors:** Na Feng, Yicong Yu, Tiecheng Wang, Peter Wilker, Jianzhong Wang, Yuanguo Li, Zhe Sun, Yuwei Gao, Xianzhu Xia

**Affiliations:** 1 College of Animal Science and Technology, Jilin Agricultural University, Changchun, 130118, People’s Republic of China; 2 Military Veterinary Research Institute of Academy of Military Medical Sciences, Changchun, 130122, People’s Republic of China; 3 Changchun Veterinary Research Institute, Chinese Academy of Agricultural Sciences, Changchun, 130122, People’s Republic of China; 4 Department of Microbiology, University of Wisconsin-La Crosse, La Crosse, Wisconsin, 54601, USA; 5 College of Animal Science, Henan Institute of Science and Technology, Xinxiang, 453003, People’s Republic of China; 6 National Research Center for Veterinary Medicine, Luoyang, 471000, People’s Republic of China; 7 Jiangsu Co-innovation Center for Prevention and Control of Important Animal Infectious Diseases and Zoonoses, Yangzhou, 225009, People’s Republic of China

## Abstract

We report an outbreak of canine distemper virus (CDV) infection among endangered giant pandas (*Ailuropoda melanoleuca*). Five of six CDV infected giant pandas died. The surviving giant panda was previously vaccinated against CDV. Genomic sequencing of CDV isolated from one of the infected pandas (giant panda/SX/2014) suggests it belongs to the Asia-1 cluster. The hemagglutinin protein of the isolated virus and virus sequenced from lung samples originating from deceased giant pandas all possessed the substitutions V26M, T213A, K281R, S300N, P340Q, and Y549H. The presence of the Y549H substitution is notable as it is found at the signaling lymphocytic activation molecule (SLAM) receptor-binding site and has been implicated in the emergence of highly pathogenic CDV and host switching. These findings demonstrate that giant pandas are susceptible to CDV and suggest that surveillance and vaccination among all captive giant pandas are warranted to support conservation efforts for this endangered species.

The giant panda is native to China and categorized as endangered (criteria C2a(i)) on the International Union for Conservation of Nature’s Red List of Threatened Species. The State Forestry Administration of China reported in the fourth national panda survey that there are 1,864 wild pandas in China, representing a 16.8% increase over the last decade^1^. The current global population of captive giant pandas is 394, and has been gradually approaching the population development goal of 500^2^. Restricted or degraded habitat poses a significant threat to wild panda populations, and wild panda habitat and protected areas have significantly expanded to include a total of 5.94 million hectares^1^. Changes in the population or habitat of giant pandas may place animals at increased risk of infectious disease and hinder conservation efforts.

Canine distemper virus (CDV) has been reported to cause morbidity and mortality in giant pandas. In 1997, three giant pandas at the Chongqing Zoo were infected with CDV, as confirmed by recovery and sequencing of the CDV hemagglutinin (H) gene from affected pandas without an effort to recover CDV isolates or characterize other viral genes^3^. Additionally, serological data from the Wolong Research Center showed that four of sixty-seven unvaccinated giant pandas had detectable CDV antibody titers^4^. These reports indicate that CDV can infect giant pandas, though it has been nearly 20 years since the last clinical cases were reported.

CDV is an enveloped, single-stranded RNA virus of the *Morbillivirus* genus and family *Paramyxoviridae*. The most common mode of CDV transmission is through aerosolization of respiratory exudate and other body excretions containing virus. CDV infections have been observed in the order Carnivora (e.g., *Canidae*, *Felidae*, *Mustelidae*, *Ursidae*, and *Procyonidae* families), as well as in nonhuman primates^5^,^6^,^7^. The recognized host range of CDV has expanded in recent years^8^. Several fatal outbreaks of CDV have been reported in captive wild populations, including lions (*Panthera leo*), tigers (*Panthera tigris*), leopards (*Panthera pardus*)^9^, African wild dogs (*Lycaon pictus*)^10^, as well as in free-ranging, wild populations of endangered species such as African wild dogs^11^, Iberian lynx (*Lynx pardinus*)^12^ and wild Amur Tigers (*Panthera tigris altaica*)^13^. Outbreaks in cynomolgus monkeys (*Macacca fascicularis*) and rhesus monkeys (*Macaca mulatta*) have been also reported^14^,^15^. Here, we report six confirmed cases of CDV infection among a group of twenty-two giant pandas in the Shanxi Rare Wild Animal Rescue and Research Center in China.

## Results

### Fatal CDV infection in giant pandas

On December 3, 2014, an eight-year old giant panda named Chengcheng presented with jaw trembling and violent convulsions of the limbs. Over the ensuing fourteen weeks, four additional giant pandas housed in the same room or adjacent rooms began to display clinical signs including mucopurulent ocular discharge, nasal and footpad hyperkeratosis, and violent convulsions of the limbs (clinical onset dates are listed in Table 1). Nucleic acids isolated from nasal swabs, urine, feces and blood collected from affected pandas at the time of clinical presentation all tested positive for CDV by RT-PCR. PCR-based tests were negative for other virus previously isolated from giant pandas (canine coronavirus) or viruses regarded as a potential threat to giant pandas (canine adenovirus, canine herpesvirus, and canine parainfluenza virus)^4^,^16^,^17^. CDV-positive giant pandas were monitored and treated with antiserum therapy.

Each of the five CDV-infected giant pandas that displayed clinical signs of infection died 7–34 days following disease onset (Table 1). No CDV serum neutralizing (SN) antibodies were detected in the five infected giant pandas showing clinical signs of CDV infection prior to death (Table 1). However, CDV RNA was detected by RT-PCR in heart, liver, spleen, lung, kidney, intestines and brain of four deceased giant pandas (Table 2). In addition, CDV RNA was detected by RT-PCR from blood and nasal swab samples collected from an asymptomatic giant panda named Zhuzhu, who was previously vaccinated against CDV in 2012 and had high-titer SN antibodies (Table 1). None of the additional sixteen giant pandas in the Shanxi Rare Wild Animal Rescue and Research Center tested positive for CDV by RT-PCR. Uninfected pandas within the Shanxi Rare Wild Animal Rescue and Research Center were placed in isolation on December 26, 2014 and vaccinated with a canarypox-vectored CDV vaccine.

### Histopathological analysis

**Figure 1:**
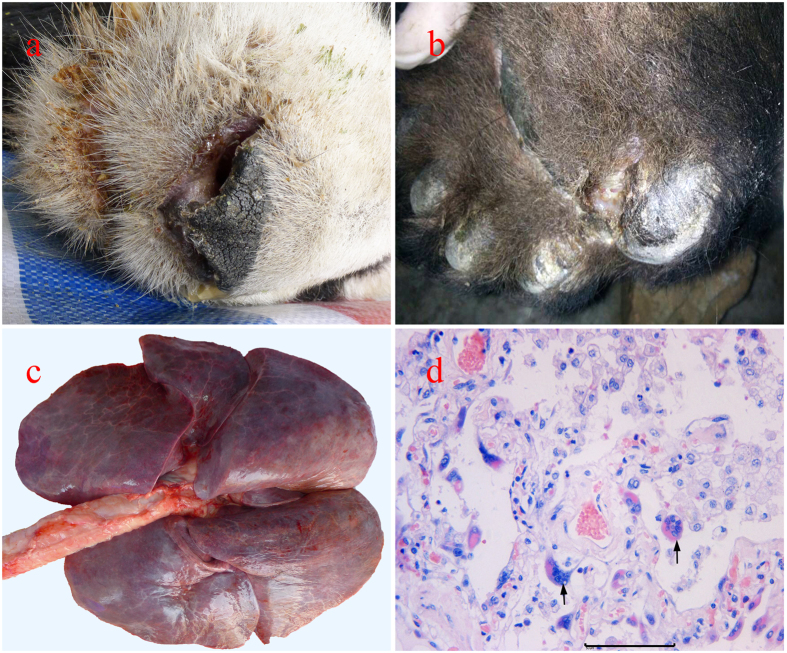
Representative clinical signs and pathological changes among CDV-infected giant pandas (*Ailuropoda melanoleuca*), China, 2014. CDV-infected pandas showed signs of (**a**) nasal hyperkeratosis, (**b**) footpad hyperkeratosis, (**c**) severe pneumonia with dark-red congestion and lungs covered with small white patches on the surface, and (**d**) interstitial pneumonia with congestion, multinuclear macrophage infiltration in the alveoli (arrows), and widening of alveolar septum. Fixed tissue samples were stained with hematoxylin and eosin (original magnification ×400, scale bar indicates 50 μm).

Late in the course of infection, giant pandas exhibited clinical signs of nasal hyperkeratosis (Fig. 1a) and footpad hyperkeratosis (Fig. 1b) which are characteristic of CDV infection in other animals^18^. Severe pneumonia with dark-red congestion was observed in affected lungs along with small white patches on the surface of the lungs of one CDV-infected giant panda named Fengfeng (Fig. 1c). Lung samples from Fengfeng were examined for histopathological analysis, whereas other samples (e.g ., brain, spleens) were not assessed due to cellular autolysis of tissues collected at necropsies performed >12 hours of after death. Histological analyses of lung tissue from Fengfeng showed interstitial pneumonia with congestion, multinuclear macrophage infiltration in the alveoli, and widening of alveolar septa (Fig. 1d). Histological observations were consistent with those previously reported in the lungs of cynomolgus monkeys and red fox (*Vulpes vulpes*) infected by CDV^14^,^19^.

### Phylogenetic Analysis

**Figure 2:**
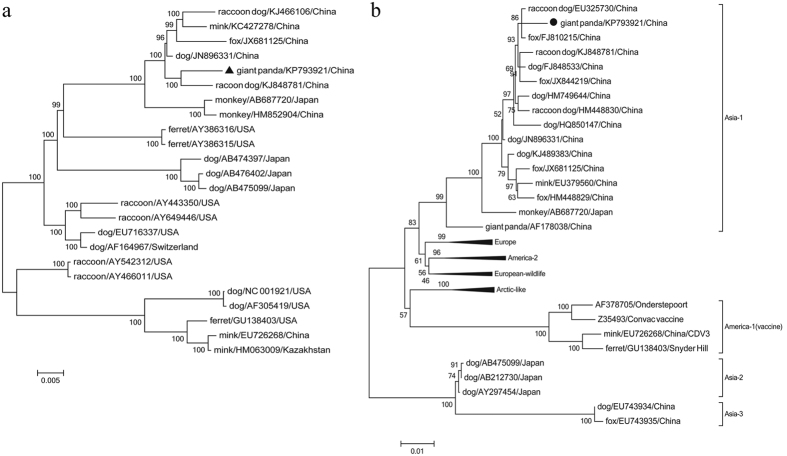
Phylogenetic trees displaying the relatedness of the giant panda CDV isolate with other CDV isolates. (**a**) Phylogenetic tree generated using complete genomic sequences. (**b**) Phylogenetic tree generated using H gene nucleotide sequences. Horizontal branch lengths are proportional to genetic distances. The black triangle and dot indicate the CDV isolate from a giant panda in China (giant panda/SX/2014). Scale bar indicates nucleotide substitutions per site.

The complete viral genome of the CDV isolate giant panda/SX/2014 was sequenced (Gen Bank accession no. KP793921) and showed highest nucleotide identity to the PS strain of CDV isolated from a dog (98.7% identity, Gen Bank accession no. JN896331) and the CDV-RD-JL strain of CDV isolated from a raccoon dog (*Nyctereutes procyonoides*, 98.6% identity, Gen Bank accession no. KJ848781) (Fig. 2a). Phylogenetic analysis and multiple sequence alignments based on the H gene sequence revealed that giant panda/SX/2014 belongs to the Asia-1 cluster. The H gene sequence of giant panda/SX/2014 was 99.1% identical to the SD(08)1 and LN(07)1 CDV strains (Gen Bank accession no. FJ810215 and EU325730) isolated from a fox and raccoon dog in Shandong and Liaoning province in China, respectively (Fig. 2b). The giant panda/SX/2014 H gene sequence showed 95.9% identity to a sequence previously recovered from a CDV-infected giant panda during an outbreak at the Chongqing Zoo in 1997 (Gen Bank accession no. AF178038) (Table 3).

### Unique features of the H protein of giant panda/SX/2014

The predicted H protein amino acid sequence of giant panda/SX/2014 was identical to H protein amino acid sequences predicted following RT-PCR and sequencing of the H gene of CDV contained in the lung samples of all deceased giant pandas. All H gene sequences were predicted to encode five unique amino acid substitutions in the H protein which have not previously been identified in other CDV isolates belonging to the Asia-1 cluster, including V26M, T213A, K281R, S300N, and P340Q. Additionally, all H gene sequences also encoded a histidine (H) residue at amino acid position 549, which has previously been associated with CDV isolates from non-canid hosts^20^. The Y549H substitution is involved in H protein binding to the signaling lymphocytic activation molecule (SLAM) receptor and has been hypothesized to contribute to the emergence of highly pathogenic CDV and host range expansion (Table 3).

## Discussion

Here, we have documented CDV infection in six captive endangered giant pandas during an outbreak in the Shanxi Rare Wild Animal Rescue and Research Center in China. Five of six CDV-infected giant pandas died as a result of the infection. Affected giant pandas presented with clinical illness beginning the first week of December, 2014 through March, 2015. All affected giant pandas were housed in the same room or adjacent rooms, suggesting that CDV may have been transmitted between pandas via direct contact and/or respiratory droplets. The surviving panda tested positive for CDV by RT-PCR, but did not develop overt clinical signs of CDV infection and was previously vaccinated against CDV, strongly supporting the utility of CDV vaccination in giant pandas.

CDV transmission to captive animals may potentially occur via direct or indirect contact with infected domestic dogs or wild carnivores. Domestic dogs were considered the likely source of infection for canine distemper in Serengeti lions in 1994^21^. In North America, wild raccoons (*Procyon lotor*) were thought to be the source of CDV infection in large captive cats in 1991 and 1992^9^. In Japan, raccoon dogs were considered to be the source of outbreak for canine distemper in tigers in 2009 and 2010^22^. In Denmark, it was speculated that wildlife species, such as foxes, raccoon dogs, and ferrets were the sources of CDV infection of farmed mink (*Neovison vison*)^23^. In China, CDV infection has been observed in domestic dogs, wild canids (fox, raccoon dogs), and non-canids (mink, monkey) demonstrating the remarkable ability of this pathogen to cross species barriers^15^,^24^. The reservoir source of CDV leading to the outbreak among giant pandas remains unclear. While there were no carnivores in the Shanxi Rare Wild Animal Rescue and Research Center, it is possible that domestic dogs or other susceptible wild animals carrying CDV in the area were responsible.

The highly variable nature of H gene sequences among viruses belonging to the *Morbillivirus* genus has been exploited to characterize CDV field strains and investigate relationships among various strains. Sequencing of CDV from giant pandas revealed five unique amino acid changes (V26M, T213A, K281R, S300N, P340Q) encoded by the H gene that have not been observed previously in Asia-1 strains. Amino acid residues at positions 549 in the CDV H protein are implicated in CDV host range restriction and pathogenesis^25^,^26^. Notably, the H gene of CDV from giant pandas possessed a Y549H substitution which has been associated with the emergence of highly pathogenic CDV and host range expansion. Before this outbreak, the Y549H substitution had only observed in twelve CDV strains isolated from mink, fox and raccoon dogs in Shandong province and an isolate from a mink in Liaoning province in China^24^. While the Shanxi Rare Wild Animal Rescue and Research Center is located a significant distance away from these provinces (>900 kilometers), these species may represent potential sources of CDV leading to this outbreak among giant pandas. Previous work has shown that serial passage of dog-derived CDV strain 5804 in ferrets led to acquisition of the Y549H substitution^27^. CDV isolates with a histidine residue at position 549 also showed enhanced virulence in raccoons relative to strains lacking histidine at this position^28^. We therefore speculate that the high-level of virulence associated with giant panda/SX/2014 infection of giant pandas may be related to the presence of a histidine residue at position 549 of the H protein. The additional unique H protein amino acid substitutions identified may reflect adaptive changes facilitating CDV infection of giant pandas. As an RNA virus, CDV is capable of rapid mutation leading to viral variants that are potentially better equipped for replication in giant pandas, as has been documented for the emergence of the Y549H substitution during serial passage in ferrets^27^.

Vaccination represents an effective approach to prevent CDV infection of domestic dogs and may have utility in captive giant panda populations. Currently, there are no standard vaccination strategies in place for the prevention of infectious diseases in captive giant pandas in China. The effectiveness of CDV vaccination in giant pandas is supported by the observation that the single panda to survive CDV infection during this outbreak (Zhuzhu) was previously vaccinated against CDV and had high-titer SN antibodies. This animal did not display clinical signs despite recovery of CDV genomic material from blood and nasal swab samples, suggesting that the protective immune responses elicited by CDV vaccination were not sufficient to prevent naturally-acquired CDV infection but may have attenuated disease. Ultimately, universal CDV vaccination of captive giant pandas may be warranted but must be also weighed against potential vaccination risks. Additional studies to better understand the safety and efficacy of CDV vaccines in giant pandas are needed as CDV vaccines are more widely implemented. In a study involving two giant pandas, a commercially available canarypox-vectored CDV vaccine safely elicited SN antibody titers above a level considered to be protective against CDV disease^29^. Due to the limited supply of the canarypox-vectored CDV vaccine and the potential risks associated with live-attenuated CDV vaccines, most giant pandas in the Shanxi Rare Wild Animal Rescue and Research Center and other organizations involved in giant panda breeding programs are not routinely vaccinated. The documentation of a CDV outbreak among captive giant pandas in China suggests that heightened surveillance and CDV vaccination should be considered in all facilities housing captive giant pandas for successful conservation of this endangered species.

## Methods

### Ethics statement

The protocol of the study was carried out in accordance with guidelines of animal welfare of World Organization for Animal Health. All experimental protocols were approved by the Review Board Military Veterinary Research Institute of the Academy of Military Medical Sciences.

### Virus detection by PCR and RT-PCR

Nasal swab, urine, fecal and blood samples from each affected panda were collected at the time of clinical disease onset. Viral DNA and RNA were isolated from samples using the AxyPrep Multisource Genomic DNA Miniprep kit (AXYGEN, Union City, USA) and RNeasy Mini kit (QIAGEN, Germantown, MD) according to manufacturer’s protocols. Extracted nucleic acids were tested by RT-PCR for CDV using primers specific for CDV H gene (P1:5′-CGAGTCTTTGAGATAGGGTT-3′ and P2: 5′-CCTCCAAAGGGTTCCCATGA-3′). RT-PCR and PCR testing for other viruses threatening giant pandas (canine adenovirus, canine herpesvirus, canine coronavirus, and canine parainfluenza virus) were performed using previously reported methods^17^,^30^. RT-PCR testing for CDV was also performed on samples collected from the heart, liver, spleen, lungs, kidneys, intestines, and brain of each deceased giant panda, with the exception of Chengcheng for whom tissue samples were not available. Serum samples were collected from the giant pandas during the outbreak to measure SN antibody titers against CDV.

### Histopathological analysis of giant pandas infected with CDV

Necropsies were performed on all deceased giant pandas at which time tissue samples were collected for histologic examination. Lung samples from the giant panda named Fengfeng, who experienced a long illness duration, were selected for histologic review using routine methods. Tissue samples were fixed in 10% phosphate-buffered formalin, embedded in paraffin wax, sectioned, and stained with hematoxylin and eosin prior to analysis.

### Virus isolation and sequencing

CDV was isolated from lung and spleen tissue collected from the deceased giant panda Dabao as follows. Tissue samples were inoculated onto monolayers of Vero cells expressing canine SLAM protein, which have been previously used to grow and isolate CDV^31^. The presence of CDV was confirmed by RT-PCR detection using primers specific for the CDV N gene (P3: 5′-GTGACTGCTCCTGATACTGC-3′ and P4: 5′-ACCAACTCCCATAGCATAAC-3′). The giant panda CDV isolate was named as giant panda/SX/2014. The complete H gene of the giant panda/SX/2014 isolate and virus contained in lung samples from deceased giant pandas was amplified for sequencing by RT-PCR using H gene specific primers (P5: 5′-GTCTTGCCTGATTGTCAGGC-3′ and P6: 5′-GGTTTGATTCAATCGTCGG-3′). The entire genome of the giant panda/SX/2014 CDV isolate was amplified and sequenced using a set of fifteen primer pairs to generate overlapping PCR amplicons (Table S1).

### Phylogenetic analysis

Phylogenetic trees were constructed using molecular evolutionary genetics analysis MEGA 6 software (http://www.megasoftware.net/mega.php) with the neighbor-joining (NJ) method to calculate distance. Bootstrapping with 1,000 replicates was performed to determine the percentage reliability for each internal node.

## Additional Information

**How to cite this article**: Feng, N. *et al*. Fatal canine distemper virus infection of giant pandas in China. *Sci. Rep*. **6**, 27518; doi: 10.1038/srep27518 (2016).

## Supplementary Material

Supplementary Information

## Figures and Tables

**Table 1:** Fatal canine distemper virus infection among giant pandas in China.

Animal	Clinical onset date	Clinical signs	Results of RT-PCR of blood andnasal swab samples	Antibodytiters#	InfectionResult	Illnessduration
Chengcheng	December 3, 2014	Strong convulsions	+	−	Dead	7 d
Dabao	December 24, 2014	Strong convulsions	+	−	Dead	12 d
Fengfeng	January 2, 2015	Strong convulsions	+	−	Dead	34 d
Xinxin	January 10, 2015	Mild convulsions	+	−	Dead	14 d
Longlong	March 14, 2015	Strong convulsions	+	−	Dead	26 d
Zhuzhu	N/A	None	+	128	Survived	N/A

N/A, not applicable.

^#^Serum neutralization (SN) antibodies against canine distemper virus.

**Table 2:** Detection of CDV by RT-PCR in tissues from dead giant pandas.

Tissue	heart	liver	spleen	lung	kidney	intestines	brain
Dabao	+	+	++	++	+	+	ND
Fengfeng	−	−	+	+++	−	+	++
Xinxin	−	+	++	++	+	+	+++
Longlong	−	−	+	+++	−	+	++

+, weakly positive; ++, positive; +++, strong positive; −, negative; ND, Not done.

**Table 3:** Nucleotide sequence identity and amino acid differences of the CDV isolated from the giant panda as compared to other closely related isolates.

Nucleotidesequence identity#	Sequence No. (strain name and isolated species)	26	178	213	281	300	340	542	549
-----	giant panda/SX/2014	*M*	A	*A*	*R*	*N*	*Q*	I	**H**
99.1%	FJ810215 (SD(08)1, fox)	V	A	T	K	S	P	I	Y
99.1%	EU325730 (LN(07)1, raccoon dog)	*	*	*	*	*	*	*	*
98.9%	FJ848533 (BJ080326, dog)	*	*	*	*	*	*	*	*
98.7%	KJ848781(CDV-RD-JL, raccoon dog)	*	*	*	*	*	*	*	*
98.7%	JX681125 (HLJ1-06, fox)	*	*	*	*	*	*	*	*
98.7%	EU379560 (SD(07)1, mink)	*	*	*	*	*	*	*	**H**
98.6%	JX844219 (LN(12)1, fox)	*	T	*	*	*	*	*	*
98.6%	HM749644 (CDV-JT1, dog)	*	*	*	*	*	*	*	*
98.6%	HM448830 (HeB(09)1, raccoon dog)	*	*	*	*	*	*	*	*
98.6%	JN896331 (PS, dog)	*	*	*	*	*	*	*	*
98.3%	KJ489383 (NJ(12)7, dog)	*	*	*	*	*	*	*	*
98.2%	HQ850147 (GS0812-4, dog)	*	*	*	*	*	*	*	*
97.7%	HM448829 (SD(09)1, fox)	*	*	*	*	*	*	*	**H**
97.0%	AB687720 (CYN07-dV, monkey)	*	*	*	*	*	*	F	*
95.9%	AF178038 (giant panda)	*	G	*	*	*	*	*	*
89.9%	AF378705(Onderstepoort)	*	G	*	*	*	*	*	**H**
89.9%	Z35493 (Convac)	*	G	*	*	*	*	*	**H**
89.9%	EU726268 (CDV3, mink)	*	G	*	*	*	*	*	**H**
89.9%	GU138403 (Snyder Hill, ferret)	*	G	*	*	*	*	F	*

^#^Percent identity in hemagglutinin nucleotide sequence of the CDV isolated from the giant panda when compared to other closely related isolates.

^*^Indicates residue identical to that of FJ810215 (SD(08)1). Italics indicates mutated residues of giant panda/SX/2014 when compared to other strains. Bold highlights isolates with a histidine residue 549 of the CDV H protein.
