# Supplementary material for: Fatal canine distemper virus infection of giant pandas in China
Source: Sci Rep. 2016 Jun 16;6:27518. doi: 10.1038/srep27518 (PMC4910525; doi:10.1038/srep27518)
Supplement: Supplementary Information [file srep27518-s1.pdf]

Supplementary data

Manuscript title:

Fatal canine distemper virus infection of giant pandas in China

Author list:

Na Feng, Yicong Yu, Tiecheng Wang, Peter Wilker, Jianzhong Wang, Yuanguo Li, Zhe Sun, Yuwei Gao, and Xianzhu Xia

Table S1 primers used to amplify the CDV genome of the giant panda/SX/2014

Primer	Nucleotide sequence (5'-3')	Position
1F	ACCAGAAAAAGTTGGCTATGGA	1-22
1R	GTCCTCCGTTGTCTTGGATGCTA	1297-1319
2F	TTCGGTAGATCCTACTTTGATC	1161-1182
2R	CGGAATAATCTTCGCCAGAATCC	2259-2281
3F	GTGAAGAGGTTAAGGGAATCGAA	2150-2172
3R	TCATCTGGTAGAACTCGTTTAGA	3279-3301
4F	TCTAGCTCGGCAATCGGATAC	3136-3156
4R	GGGATCCTGGACAATGCCACCAA	4472-4494
5F	GATGTACTGGTAAGATGAGC	4213-4232
5R	TATGACCAAGTACTGGTGACT	5418-5438
6F	CAGATACATTGGAATAATTTGTC	5340-5362
6R	GCTTTCGTATACCATATCAGGG	6581-6602
7F	GGCAACAAGTTTATTCTGTCA	6396-6416
7R	GAAGTAGTAGCTCCACTGCATCT	7637-7659
8F	TTAGCAGATTGCTGAAAGAGG	7287-7307
8R	TCAGGTCTACCCTTGGTAGTT	8737-8757
9F	CGGTTGACATTACCTCTAGATC	8300-8321
9R	CATGGTCTCATAGGAGACAACT	10163-10184
10F	ACAGGACAATGGCTTCTATACTG	9977-9999
10R	GACCAGGTGCTTGGAACTCT	11373-11392
11F	AGTACTGTCTGAATTGGCG	11026-11044
11R	ATAGTTATGAGAGCCTGATGCGC	12568-12590
12F	CAGGATGAGGATGAAGCTT	12144-12162
12R	ATGGGTATTACGCAACAATCTG	13129-13150
13F	GCACGTTATGCAACAATCTCGA	12951-12972
13R	TTCCATGTCAGACCAACATG	13983-14002
14F	GTGATGAGGATGTCATACCTG	13813-13833
14R	TTACTAACACTGACCCTACC	14756-14775
15F	GGAGTGTTGATTGTTACAAG	14602-14621
15R	ACCAGACAAAGCTGGGTATGA	15670-15690
